# Commentary: Identification of diagnostic genes for myocardial ischemia reperfusion injury associated with metabolic syndrome through the integration of bioinformatics analysis, molecular docking and experimental validation

**DOI:** 10.3389/fimmu.2026.1968659

**Published:** 2026-09-29

**Authors:** Yifei Zhang, Wuxiao Yang, Xuewen Li

**Affiliations:** 1 Department of Cardiology, Third Hospital of Shanxi Medical University, Shanxi Bethune Hospital, Shanxi Academy of Medical Sciences, Tongji Shanxi Hospital, Taiyuan, China; 2 Department of Geriatrics, Shanxi Provincial People’s Hospital, Taiyuan, China

**Keywords:** bioinformatics, diagnostic biomarkers, drug discovery, machine learning, metabolic syndrome, myocardial ischemia-reperfusion injury

## Introduction

We read with interest the study by Liu et al., which investigated diagnostic biomarkers and therapeutic strategies for myocardial ischemia-reperfusion injury (MIRI) in the context of metabolic syndrome (MetS) (1). Through bioinformatics analysis, machine learning, molecular docking, and cellular experiments, the authors identified five hub genes DAK, GTF3C5, KCNMB1, TRAF1, and ZNF692 and constructed a diagnostic model with AUC values from 0.875 to 0.969. They also demonstrated dexamethasone’s potential protective effect via molecular docking and H9C2 cell assays. Given the widespread use of percutaneous coronary intervention and the limited options for MIRI, novel biomarkers are urgently needed. The study offers valuable insights into MIRI detection and drug discovery. However, upon careful reading, we noted several methodological issues that may affect the generalizability of the findings. We offer these observations for consideration by the authors and readers, in the hope of facilitating future research.

## Definition of the normal control group

MIRI is a pathophysiological continuum in which reperfusion injury amplifies ischemic damage, driven by oxidative stress, calcium overload, mitochondrial dysfunction, inflammation, apoptosis, and autophagy dysregulation (2). It is not merely ischemia plus reperfusion, but a distinct entity with unique molecular signatures.

In Liu et al., the GSE6381 dataset comprised 12 right ventricular specimens from pediatric ventricular septal defect repair surgery, collected at three time points: Stage 1 (post-cardioplegic arrest), Stage 2 (end-ischemia), and Stage 3 (5 minutes post-reperfusion). The authors combined Stages 1 and 2 as the control group and designated Stage 3 as the MIRI group. The concern with this grouping is that the control group does not represent a normal baseline but rather two distinct time points of myocardial ischemia. According to the GEO database record, Stage 1 samples were taken “immediately after cardiac arrest following administration of standard hyperkalemic, cold blood cardioplegia,” and Stage 2 samples were taken “at 50 minutes of cardiac arrest”—both unequivocally representing ischemic conditions. The original study by Arab et al. (3) was designed as a time-series analysis to capture transcriptional dynamics during intraoperative ischemia-reperfusion, with each patient serving as their own control, and at no point was any time-point designated as “normal.”

Liu et al. therefore compared two sequential pathological states, ischemia versus ischemia plus reperfusion, rather than MIRI versus true normal baseline. This approach has direct implications for the identification of MIRI-associated genes. Ischemic stress itself triggers extensive transcriptional reprogramming. Consequently, the observed differences between the two groups may reflect the progression from ischemia to reperfusion rather than reperfusion-specific injury, potentially masking genes that are specifically altered by reperfusion. In addition, genes altered by surgical manipulation or cardiopulmonary bypass could be misidentified as MIRI-related. The unequal sample sizes between the two groups (8 versus 4 samples) may also affect the robustness of statistical comparisons. In preclinical models and hypoxia/reoxygenation experiments, the standard control is the normal (sham or untreated) state, against which the complete MIRI process is compared. Thus, the grouping strategy differs from established MIRI research frameworks and should be considered when interpreting the conclusions.

## Sample size and risk of model overfitting

In the GSE6381 dataset, the MIRI group comprised four samples (Stage 3, n = 4), on which the authors performed LASSO and SVM-RFE feature selection and constructed a five-gene diagnostic model, reporting an AUC of 1.0. Several methodological considerations are worth noting when interpreting this result. First, when sample sizes are small, the performance estimates of machine learning models can be influenced by the specific training data, and even with cross-validation, they may yield optimistically high results (4). Second, feature selection methods such as LASSO and SVM-RFE may be sensitive to random fluctuations in the data when the sample size is limited, so the stability of the selected feature genes needs to be further evaluated in larger cohorts. Additionally, the study did not clearly report whether the reported AUC was obtained from an independent test set, cross-validation, or the same samples used for feature selection, and this information is crucial for assessing the model’s generalizability. Furthermore, the appearance of pathways such as viral carcinogenesis and transcriptional dysregulation in cancer within the PPI network, which have limited relevance to the known pathophysiology of MIRI, also warrants further investigation in future studies. These factors do not necessarily mean the model is invalid, but they suggest that before translating this diagnostic model into clinical application, its performance should be validated in larger, independent human MIRI cohorts.

## Tissue origin and cross-species validation strategy

Two additional methodological considerations merit brief mention. First, the MIRI-associated differentially expressed genes were derived from human cardiac tissue, whereas the MetS-associated DEGs were obtained from human peripheral blood mononuclear cells (PBMCs). The overlap analysis of genes from these two distinct tissue sources may introduce considerable tissue-specific background signals. Although previous studies have reported gene expression overlap between PBMCs and cardiomyocytes, and some overlapping genes may genuinely reflect shared molecular mechanisms (5, 6), others may represent tissue-specific expression patterns that do not translate across organs. Therefore, the biological relevance of these overlapping genes should be interpreted with caution.

Second, the hub genes identified from the human dataset were validated directly in mouse MIRI datasets, without an intermediate validation step in an independent human MIRI cohort. It is important to distinguish between the limitations of cross-species validation and evidence that contradicts the original findings. Mouse models have been indispensable for defining the mechanisms of cardiac injury, and the validation results from mouse cardiac tissue provide supportive mechanistic evidence at the organ level. However, species-specific differences between humans and mice in molecular responses to myocardial ischemia-reperfusion injury limit their utility in fully recapitulating human MIRI (7). Therefore, while mouse validation offers valuable mechanistic insights, it cannot substitute for validation in independent human cohorts. We suggest that future studies prioritize validation of hub gene expression in independent human cardiac tissue samples, such as endomyocardial biopsies or surgical specimens. Specifically, control groups should be selected to represent a true normal baseline, findings should be validated in independent human cohorts, and sample size requirements for machine learning models should be carefully considered. When evaluating model performance in small datasets, strategies such as independent test sets and external validation should be employed to establish more robust evidence for clinical translation.

## Discussion

Liu et al. have made a valuable exploratory effort in identifying biomarkers and therapeutic candidates for MetS-associated MIRI, and the five hub genes and dexamethasone data merit further attention. However, the methodological concerns outlined above, particularly the control group definition, tissue origin, and cross-species validation strategy, may affect interpretation and clinical translation. We suggest that future studies revisit the GSE6381 grouping strategy and validate the findings in larger independent human cohorts to better define clinical utility.

MIRI involves dynamic interactions among multiple cell types, including cardiomyocytes, endothelial cells, smooth muscle cells, fibroblasts, and macrophages. Future studies could employ single-nucleus RNA sequencing to resolve cell-type heterogeneity in cardiac tissue without the dissociation artifacts of conventional single-cell methods, thereby providing a more faithful view of the myocardial transcriptome. Spatial transcriptomics could further reveal the spatial organization of gene expression and intercellular communication (8). Virtual knockout and overexpression could predict the functional roles of target genes in specific cell types, while virtual drug screening, molecular docking, and molecular dynamics simulations could assess the binding modes and stability of small-molecule compounds with target proteins (9), refining candidate molecules for subsequent validation. Together, these computational strategies may accelerate the translation from biomarker discovery to therapeutic intervention. We believe the analytical framework and candidate genes provided by this study will serve as a useful foundation for subsequent work.
